# Lignin-Derived Quinone Redox Moieties for Bio-Based Supercapacitors

**DOI:** 10.3390/polym14153106

**Published:** 2022-07-30

**Authors:** Jincy Parayangattil Jyothibasu, Ruei-Hong Wang, You-Ching Tien, Chi-Ching Kuo, Rong-Ho Lee

**Affiliations:** 1 Department of Chemical Engineering, National Chung Hsing University, Taichung 402, Taiwan; jincycusat@gmail.com (J.P.J.); see10321312@gmail.com (R.-H.W.); poweramoney@googlemail.com (Y.-C.T.); 2 Research and Development Center of Smart Textile Technology, Institute of Organic and Polymeric Materials, National Taipei University of Technology, Taipei 10608, Taiwan; kuocc@mail.ntut.edu.tw

**Keywords:** hydroquinone, lignin, quinone, redox electrolytes, supercapacitor electrodes

## Abstract

Because of their rapid charging and discharging, high power densities, and excellent cycling life stabilities, supercapacitors have great potential for use in electric vehicles, portable electronics, and for grid frequency modulation. The growing need for supercapacitors that are both efficient and ecologically friendly has generated curiosity in developing sustainable biomass-based electrode materials and electrolytes. Lignin, an aromatic polymer with remarkable electroactive redox characteristics and a large number of active functional groups, is one such candidate for use in renewable supercapacitors. Because its chemical structure features an abundance of quinone groups, lignin undergoes various surface redox processes, storing and releasing both electrons and protons. Accordingly, lignin and its derivatives have been tested as electroactive materials in supercapacitors. This review discusses recent examples of supercapacitors incorporating electrode materials and electrolytes derived from lignin, focusing on the pseudocapacitance provided by the quinone moieties, with the goal of encouraging the use of lignin as a raw material for high-value applications. Employing lignin and its derivatives as active materials in supercapacitor electrodes and as a redox additive in electrolytes has the potential to minimize environmental pollution and energy scarcity while also providing economic benefits.

## 1. Introduction

Our industrial development and the rising human population are necessitating a hunt for environmentally friendly and long-term energy sources [1,2]. Although it is possible to harvest energy from the wind, sun, and water, these renewable sources generate energy in an intermittent manner [3], making energy storage increasingly important [4]. Electrochemical supercapacitors are attractive energy storage systems because of their high power densities, long cycle lives, and high efficiencies [5], but they generally have energy densities lower than those of batteries and fuel cells [6]. Therefore, a great deal of work has been done to enhance the energy densities of supercapacitors while preserving their high power densities. Supercapacitors are classified in terms of their charge storage mechanisms, mainly into electrochemical double-layer capacitors (EDLCs), pseudocapacitors, and hybrid capacitors. In an EDLC, charge storage occurs through electrostatic adsorption and desorption of electrolyte ions at the interface between the electrode and electrolyte [7]. Pseudocapacitive charge storage involves rapid and reversible Faradaic charge transfer on or near the surfaces of the electrode materials [8]. The Faradaic redox reactions occur on the surfaces of the solid electrodes and in the bulk near those surfaces; therefore, the specific capacitances and energy densities of pseudocapacitors are much larger than those of EDLCs. The redox reactions, although rapid, are limited by the rates of diffusion and the degrees of penetration of the electrolyte (cf. rapid ionic adsorption in EDLCs). Moreover, similar to batteries, pseudocapacitors often lack stability during cycling because of the redox reactions that occur at their electrodes [9]. Hybrid supercapacitors combine the benefits of EDLCs and pseudocapacitors while limiting their drawbacks and, thereby, display enhanced energy storage [10,11]. By storing charges in both Faradaic and non-Faradaic processes, the energy and power densities of hybrid supercapacitors can be much higher than those of their respective components.

The quest to deliver clean energy from renewable sources is encouraging the development and optimization of innovative materials for use in supercapacitors. For the sustainable development and widespread application of supercapacitors, it would be preferable to prepare the electrodes and electrolytes out of renewable, inexpensive, and abundant natural materials. In recent years, biomass-derived materials have become popular in supercapacitor research because of their availability, bio-renewability, bio-degradability, cost-effectiveness, and eco-friendliness. Lignin is nature’s most prevalent amorphous aromatic polymer; after cellulose, it is the second most abundant biomass. Lignin has many properties that make it suitable for application in supercapacitors: an abundance of functional groups, strong biocompatibility, biodegradability, high thermal stability, low toxicity, and a high carbon content [12,13]. Pulp and paper mills generate around 70 million tons of lignin per annum, with only about 2% being utilized commercially as, for example, additives and stabilizing agents [14]. The rest is burnt or disposed of, potentially producing pollution and damaging the environment [15]. As a result, innovative ideas are being sought for the efficient application of waste lignin. This review describes recent breakthroughs in supercapacitor technologies using lignin-based electrodes and electrolytes. We discuss various strategies for preparing lignin-based composites, their electrochemical performance, and the prospects of using lignin-derived materials in supercapacitors.

## 2. Fundamentals of Lignin

Lignocellulose is an abundant bio-resource, consisting primarily of cellulose (40–50%), hemicellulose (20–30%), and lignin (25–35%) [12]. Lignin, a major component of vascular plants, is chemically intertwined with hemicellulose and wrapped around the exterior of cellulose fibers. Lignin is the most abundant non-carbohydrate component in lignocellulosic biomass, providing plant cell walls with strength and hydrophobicity while preserving polysaccharides from microbial decomposition [16]. As a result of the many structural units, linkages, and complicated connections between lignin and glycan in the cell wall, lignin is one of the most complex natural polymers in nature.

**Figure 1:**
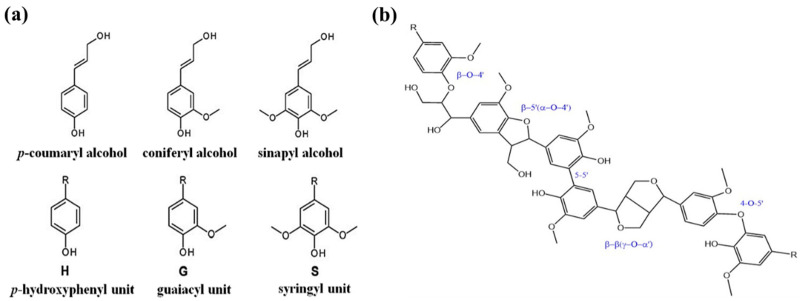
(**a**) Structures of monomeric precursors of lignin; the monolignols H, G, and S (R = lignin); and (**b**) a model lignin featuring five G units connected by five common interunit linkages. Reprinted with permission from Ref. [21]. Copyright 2020 John Wiley & Sons.

The lignin polymer contains three structural units: syringl (S), guaiacol (G), and *p*-hydroxyphenyl (H) moieties (Figure 1) [13]. Because these different monolignols are connected in a three-dimensional network through multiple linkages, making complexities in structural elucidation structural analysis of lignin is much more difficult than that of other polymers [17,18]. The structure of lignin is more akin to a “chemical web” [19], lacking a clearly defined primary structure. The plant species (e.g., softwood, hardwood, or grass), its growth environment, and its various components (e.g., stalks, leaves, or seeds) all feature lignin at different levels of content and with different structural compositions, molecular weights, and interunit linkages. In general, coniferous species (softwoods) have lignin contents (25–35%) greater than those of deciduous species (hardwoods, 15–30%) [20]. Softwood lignin features predominantly G-type units (90–95 wt.%), whereas hardwood lignin features mostly G- and S-type units [15]; all three monolignol forms may be found in grass lignin. Furthermore, different pretreatment conditions can impact the amounts and types of monolignols, making the S:G:H ratio a helpful indicator during the lignin separation process.

Lignin is divided into two types based on its origin: native or technical. Native lignin refers to the original structure of lignin in lignocellulose that has not been altered. Extraction of lignin from lignocellulose during the pulping processes changes its chemical structure and introduces inorganic contaminants; technical lignin is the name given to this kind of extracted lignin [22]. Kraft lignin (KL), organosolv lignin, enzymatic lignin, hydrolysis lignin, and pyrolytic lignin are common examples of technical lignins [23]. Because they undergo combinations of several reactions, including catalyzed biomass hydrolysis, condensation of lignin fragments, reduction of native lignin functional groups, and generation of new functional groups, technical lignins vary markedly from their native counterparts. Chemical pulping, which cleaves chemical bonds between lignins and carbohydrates at high temperatures and pressures, is currently used to produce the majority of technical lignins. The pulp and paper industry primarily uses Kraft, sulfite, soda, and organosolv pulping as a means of separating technical lignin, producing KL, lignosulfonate, soda lignin, and organosolv lignin, respectively [12], each having diverse structures, compositions, and characteristics.

Lignin can also be isolated in integrated lignocellulose biorefineries, using various biomass pretreatment methods (e.g., hydrothermal, dilute acid, and ammonia-based processes) [24]. In the biorefinery approach, the three basic components of lignocellulose (cellulose, hemicellulose, lignin) are extracted separately to achieve total utilization. Lignin is usually considered a byproduct in a biorefinery, due to its irregular structure. Among the various pretreatment methods in a biorefinery, physicochemical processes using steam explosion (SE) or mechanical grinding under high temperatures and pressures have been the most suitable for biomass fractionation, yielding high-purity lignin. Lignin recovery strategies, including lignin-first biomass fractionation procedures, have been progressing as a result of greater knowledge of the properties of lignin and its ever-increasing applications.

All pulping and biorefinery operations lead to the degradation of lignin. The reactions involved always result in a lower number of oxygenated aliphatic moieties, notably *β*-O-4 and aliphatic OH units; increases in the contents of phenolic OH, COOH, and saturated aliphatic moieties; and greater degrees of condensation (DCs), relative to those of the native lignins [13]. Nevertheless, considerable structural variations exist among the various technical lignins. The extent to which lignin degradation occurs is determined by the nature of the lignin isolation process and its severity.

### 2.1. Application of Lignin in Supercapacitors

**Figure 2:**
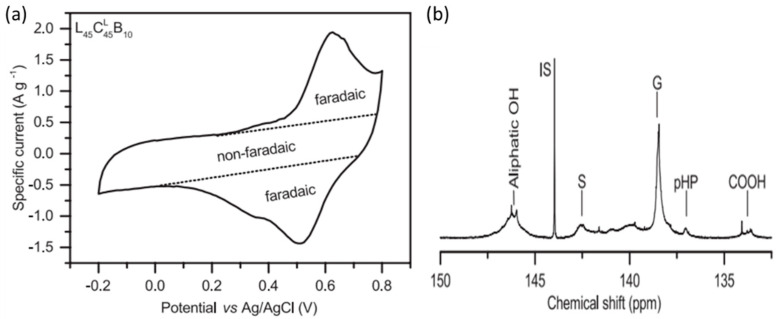
(**a**) CV trace (recorded at 5 mV s^−1^) of a lignin composite electrode in 1 M HClO_4_, with the contributions made by the Faradaic and non-Faradaic processes to the charge storage. (**b**) The 31P NMR spectrum of phosphorylated lignin, featuring signals for aliphatic OH groups; the internal standard (IS); the S, G, and p-hydroxyphenyl (pHP) groups; and COOH groups. Reprinted with permission from Ref. [27]. Copyright 2017 John Wiley & Sons.

It is difficult to develop lignin-based electrodes for energy storage devices because of its inherent insulating properties. As a result, only a few reports have appeared describing the electrochemical characteristics of lignin. Milczarek et al. reported that lignosulfonate could be electropolymerized on a glassy carbon (GC) electrode for energy storage applications [25]. From a sulfuric acid solution, they subjected lignosulfonate to oxidative electropolymerization on a GC electrode to fabricate a chemically modified electrode possessing anionic characteristics and redox activity. Repeated cycling of the lignosulfonate in an acidic electrolyte resulted in the formation of a new chemical species—quinone (Q) units—presumably through oxidation of the monolignols [26]. Because they include methoxyl (OMe) structures, the S and G monolignols can be oxidized to Q structures, but the third monolignol, H, cannot. Thus, lignin derivatives from plants having high contents of S and G units are better candidates for increasing the density of Q units in modified electrodes. To study the influence of the various lignin constituents and the function of lignin for charge storage, Liedel et al. used ball-milling and film-casting on a graphite sheet current collector to fabricate composite electrodes from Kraft lignin (KL) and conductive carbon (167 m^2^ g^−1^, C^L^) with a 10% binder [27]. Cyclic voltammetry (CV) measurements (Figure 2a) revealed considerable separation of the oxidative and reductive signals, as well as discrete redox signals and a rectangular region, demonstrating the influence of redox and electrochemical double-layer (EDL) charge storage, respectively. Figure 2a roughly displays the individual contributions to charge storage. The reversible redox couple near 0.35 and 0.55 V represents the Q/hydroquinone (QH_2_) interconversions of various groups in lignin, with the shoulder near 0.35 V representing S groups and the peak near 0.55 V representing G groups. ^31^P NMR spectroscopy (Figure 2b) revealed that the content of S groups (0.48 mmol g^−1^) in KL was much lower than that of G groups (3.29 mmol g^−1^).

Admassie et al. reported the electrochemical and supercapacitance performance of various lignin inside biopolymer-based electrodes [28]. They used ^31^P NMR spectroscopy to investigate the chemical structures of several softwood and hardwood lignin derivatives from subtropical and tropical plants (eucalyptus, acacia, and African redwood), after phosphitylation of the biopolymers. The specific capacitance and charge capacity both rose significantly upon increasing the S-to-G ratio, suggesting that Q units produced by the S units were the major contributors to the charge storage effect in these biopolymer electrodes.

With improvements in commercial manufacturing, lignin is now widely available and inexpensive, thereby lowering device manufacture costs. The most prevalent element in lignin is carbon, accounting for more than 60% of its weight. Lignin is responsible for over 30% of the biosphere’s organic carbon, suggesting its great potential as a carbon precursor. Studies of lignin in energy storage devices have increased considerably. Lignin and lignin-derived materials have several properties that make them attractive components for the manufacture of rechargeable batteries and supercapacitors. Many of the functional groups in lignin (particularly the benzyl and phenolic groups) function as active reaction sites, allowing the storage of ions in the case of supercapacitors. The various forms of lignin possess different functionalities, leading to a range of properties for energy storage. Lignosulfonates, for example, have an abundance of sulfur atoms and have been employed in rechargeable batteries and supercapacitors as sulfur-doped agents. Nevertheless, lignin cannot be used directly for electrodes because it is inherently an insulator [29]. One of the best strategies for using lignin is to develop composites with other conductive materials (e.g., conducting polymers) to increase its electrochemical performance as an electrode material.

### 2.2. Lignin-Based Electrode Materials for Supercapacitors

#### 2.2.1. Carbon Material/Lignin Composites

**Figure 3:**
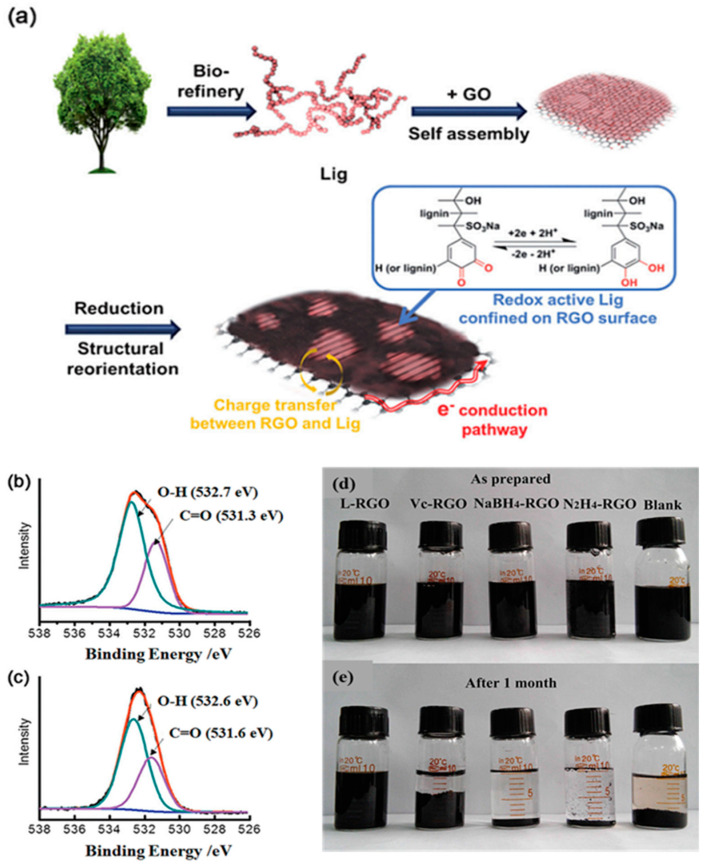
(**a**) Mechanism of energy-storage in RL composites derived from natural polymers. (**b**,**c**) High-resolution O 1s XPS spectra of RL-60 recorded (**b**) before and (**c**) after charging. Reprinted with permission from Ref. [30]. Copyright 2014 John Wiley & Sons. (**d**,**e**) Photographs of RGO dispersions treated with various reducing agents: (**d**) as-prepared and (**e**) after 1 month. Reprinted with permission from Ref. [32]. Copyright 2017 Elsevier.

Kim et al. confined lignin nanocrystals on reduced graphene oxides (RGOs) and studied their pseudocapacitive properties for sustainable energy storage materials [30]. The aromatic units on the lignin species and RGO sheets experienced strong π–π and hydrophobic interactions, forging strong connections in the RGO–lignin (RL) composites. The lignin units separated as discrete nanocrystals and were firmly confined on the surfaces of the RGOs (Figure 3a), a favorable arrangement for electrochemical processes that demand uniformly distributed and discrete electroactive nanomaterials having large surface areas and good interactions with conductive substrates. The noncovalent interactions between the lignin and the RGO sheets did not disrupt the intrinsic structure of the RGO, allowing the transfer of charge from the electron-withdrawing groups of lignin (i.e., sulfonate and carboxylate groups) to the RGO. The CV of the hybrid electrode indicated a reversible redox wave at 0.52 V. XPS spectra recorded before and after charging (Figure 3b,c) revealing the change in the chemical circumstance of the Q moieties, arising from specific interactions with protons generated through the redox reactions of the renewable hybrid electrodes (RHEs). Equation (1) represents the reversible Faradaic reaction occurring in the RL composites:

(Lignin-QH_2_)@RGO ⇔ (Lignin-Q)@RGO + 2H^+^ + 2e^−^ (1)

where the Q/QH_2_ transformation of the RGO-bound lignin could store and release two electrons and protons while charging and discharging, respectively, in aqueous acidic solutions. Thus, two electrons and two protons were stored in a structure containing 6 C atoms and 2 O atoms, giving a charge density of 2 Faraday/108 g, equal to 1787 C g^−1^ or 496 mA h g^−1^ [31]. Ye et al. demonstrated that lignin could be employed as a green reductant when preparing RGO through the deoxygenation of GO sheets, and then as a morphological guiding agent in the construction of three-dimensional (3D) RGO-derived composites [32]. Because π–π interactions with lignin hindered the aggregation of the RGO sheets, the RGO solution generated in the presence of lignin was more stable than those obtained using common reductants (e.g., ascorbic acid) (Figure 3d,e). The RL composite produced through hydrothermal carbonization had a large specific surface area, high conductivity, high specific capacitance (190 F g^−1^ at 0.5 A g^−1^), and excellent cycling stability (86.5% retention of capacitance after 10,000 continuous cycles of galvanostatic charging/discharging).

To maximize the benefits of lignin while preventing graphene sheets from aggregating, Yang et al. developed a simple one-step hydrothermal technique for fabricating lignin/graphene hydrogels [29]. Here, the lignin provided strong pseudocapacitance while preventing re-stacking of the graphene sheets. When used directly as a binder-free supercapacitor electrode, the 3D porous composite hydrogel exhibited high specific capacitance (549.5 F g^−1^) at a current density of 1 A g^−1^, as well as excellent rate capacity and cycling stability. The synergistic effect of pseudocapacitance (resulting from the reversible redox reactions of lignin) and electrical double-layer capacitance (resulting from the large specific surface area of the graphene hydrogel) contributed to the high specific capacitance of the lignin/graphene hydrogels. To overcome the low cycling efficiency and high self-discharging rate of lignin/graphene composite–based electrodes, Geng et al. developed a reconfigurable and hierarchical graphene cage—imitating the prey-trapping of a Venus flytrap—to capture lignin [33]. The reconfigurable graphene trapped the lignin within the electrode and prevented its dissolution, while also acting as a 3D current collector and providing channels for efficient electron transport throughout the electrochemical process. With its unique bio-inspired design, the graphene–lignin cathode exhibited high capacitance (211 F g^−1^ at 1.0 A g^−1^) and excellent rate performance and cycle stability (88% retention of capacity after 15,000 cycles).

**Figure 4:**
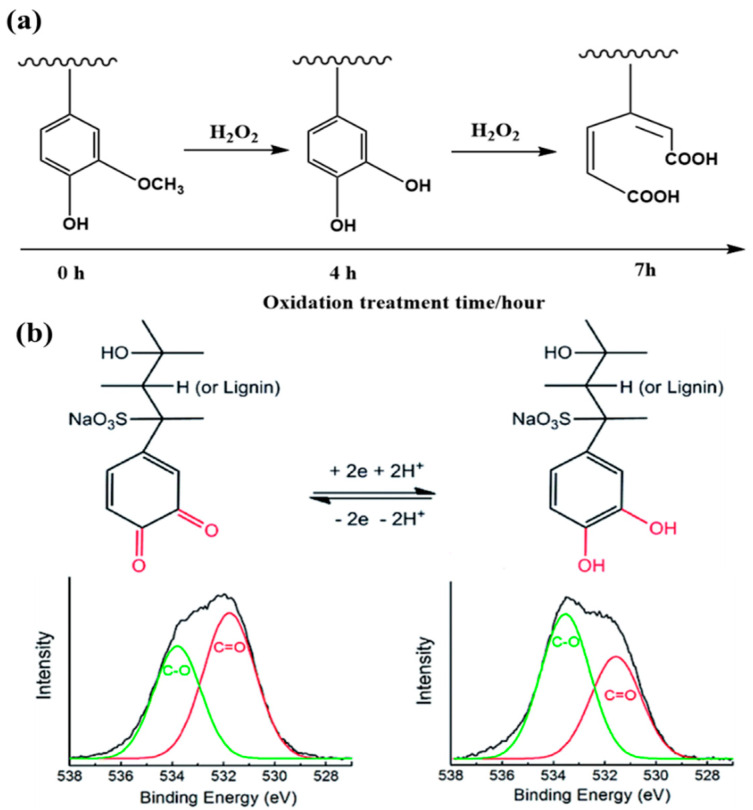
(**a**) Changes in the functional groups in KL upon oxidation. Reprinted with permission from Ref. [35]. Copyright 2020 John Wiley & Sons. (**b**) Mechanism of redox transfer of the Q/QH_2_ structure during the charge/discharge process. High-resolution O 1s XPS spectra of the LS-GH (left) before and (right) after charging. Reprinted with permission from Ref. [34]. Copyright 2017 Royal Society of Chemistry.

Although several papers describe the redox-active properties of lignin, only a few examples have appeared regarding the use of lignin-based, self-supported electrodes in flexible devices (Figure 4) [34,35]. Using lignosulfonate-functionalized graphene hydrogels (LS-GHs; LS mass ratio: ca. 29.7 wt.%), Li et al. created a new metal-free, flexible supercapacitor [34]. The LS-GHs were sufficiently mechanically sturdy for handling and cutting into slices. Because of the reversibility of the redox-based charge transfer of the Q groups in the lignin, this supercapacitor performs as well as, or better than, previously reported pseudocapacitive supercapacitors based on transition metals. An integrated flexible device featuring the LS-GH (electrodes) and H_2_SO_4_–polyvinyl alcohol (PVA) gel (electrolyte) exhibited outstanding capacitive performance (408 F g^−1^ at 1 A g^−1^; 75.4% retention of capacitance at 20 A g^−1^; 84.0% retention of capacitance after 10,000 cycles). The Q/QH_2_ structure in the conductive network allowed the storage and release of two electrons and protons during the reversible charge/discharge process. The resulting status of the oxygen atoms in the LS-GH network varied before and after charging, as evidenced through XPS analysis is shown in Figure 4b. After charging, there was observable decrease in the content of C=O groups.

Liu et al. employed a solvent-free mechanical milling process to mix graphite and lignosulfonate for the scalable production of additive-free lignosulfonate/graphene electrodes [36]. They sheared the graphite into microscopic particles with sizes ranging from 50 to 2000 nm. Graphene structures with few layers were generated during ball-milling. The conductivity and discharge capacity of the hybrid electrodes were 290 S m^−1^ and 35 mA h g^−1^, respectively. Because these electrodes exhibited a high self-discharge rate, resulting in losses of both energy density and power density, it was critical to explore the self-discharge process. Therefore, in a subsequent study, Liu et al. employed self-discharge measurements and models to better understand the mechanism of the self-discharging of the lignosulfonate/graphene electrodes, concluding that it occurred through a combination of activation control and diffusion control, depending on the charging potential [37].

**Figure 5:**
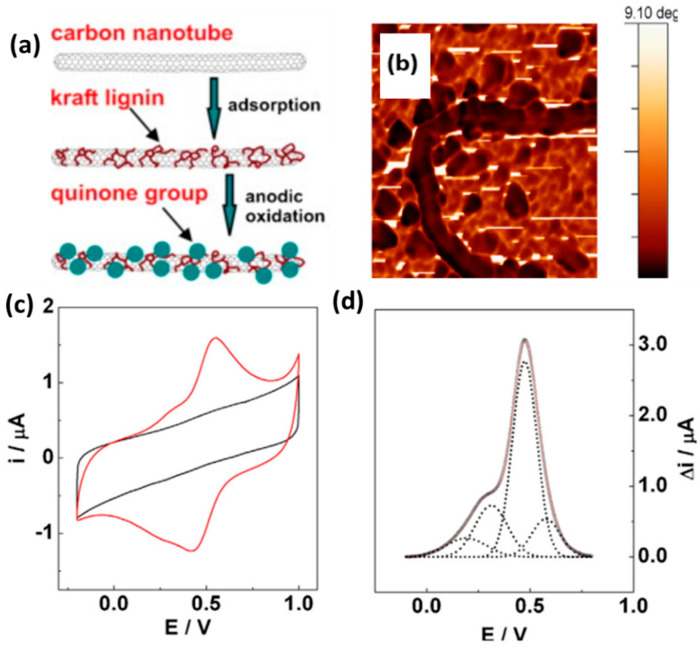
(**a**) Schematic representation of the fabrication of CNT/KL nanocomposites. (**b**) AFM phase-contrast image of KL-functionalized CNTs; image size: 0.6 µm × 0.6 µm. (**c**) CV traces of unmodified (black) and KL-functionalized (red) CNTs, recorded at 50 mV s^−1^. (**d**) Anodic differential pulse voltammogram of an CNT/KL-modified Au electrode, deconvoluted into Gaussian components. Reprinted with permission from Ref. [31]. Copyright 2013 Elsevier.

Carbon nanotubes (CNTs) interact through π-stacking with polymers containing aromatic moieties. Milczarek et al. prepared multi-walled CNTs surface-functionalized with KL and studied their electrochemical characteristics (Figure 5a) [31]. The lignin-modified nanotubes readily dispersed in an organic solvent (DMSO) and aqueous ammonia (0.1 M), yielding black solutions; such solutions did not form with unmodified CNTs. Atomic force microscopy (AFM) of the CNT/KL nanocomposite revealed debundling of the CNTs, as well as densely packed globular structures on the surfaces of the individual CNTs (Figure 5b). The redox behavior of the CNT-supported biomolecules was persistent and reversible, due to the KL-derived Q units (Figure 5c). The mass-specific capacitances (*C*_m_) of the unmodified and KL-functionalized CNTs, determined from their CV curves, were 75 and 143 F g^−1^, respectively. Figure 5d displays the anodic differential pulse voltammogram of the composite, deconvoluted into four Gaussian curves representing oxidation processes occurring at different redox centers. The complex character of the lignin suggests that these curves originated from Q units experiencing different chemical environments. Peng et al. assembled a high-performance, biomass-based, flexible, solid-state supercapacitor featuring a lignin/single-walled CNT (SWCNT_HNO3_) hydrogel as flexible electrodes and a biocompatible cellulose/Li_2_SO_4_ gel prepared using a simple phase-inversion method as an electrolyte separator [38]. The supercapacitor exhibited good specific capacitance (292 F g^−1^ at 0.5 A g^−1^), superior rate capability, and a remarkable energy density (17.1 W h kg^−1^ at a power density of 324 W kg^−1^). Moreover, the pressure-sensitive characteristics of the biomass-based, compressible supercapacitor were outstanding, with its shape being completely recoverable (i.e., without plastic deformation) after performing a compression cycle.

Using a simple ultrasonic-assisted deposition procedure, Zhou et al. prepared KL-modified, HNO_3_-treated active carbon (KL/TAC) and examined its potential as a supercapacitor electrode material [39]. The combined effects of the treatment with HNO_3_ and the coupling of KL enhanced the electrochemical properties of activated carbon (AC), with the as-prepared KL/TAC material exhibiting a specific capacitance (293 F g^−1^) significantly higher than those of the HNO_3_-treated AC (TAC, 120 F g^−1^) and the KL-modified AC (KL/AC, 118 F g^−1^). In a subsequent study, Zhou et al. used a functional group modification strategy to convert the OMe groups in KL to phenolic OH groups, using H_2_O_2_ as the oxidant and Fe_2_O_3_ as the catalyst, thereby facilitating the formation of additional QH_2_ units and leading to higher pseudocapacitances [35]. This mild H_2_O_2_ system allowed the variable oxidation of KL. The highest abundance of phenolic OH groups was present in the oxidized KL (OKL) sample subjected to 4 h of oxidation. Excessive oxidation (>5 h) resulted in the transfer of phenolic OH groups to unwanted products. Compared with the raw KL, the OKL materials had a maximum increase of 25.6% in their content of phenolic OH groups. Composite electrode materials were formed from the TAC mixed with the OKL. The OKL/TAC composite electrode exhibited a specific capacitance of 390 F g^−1^ at 0.5 A g^−1^–21.9% higher than that of the KL/TAC system (322 F g^−1^) and double that of the single-phase TAC system (132 F g^−1^). Zhou et al. further improved the electrochemical performance of the lignin-based composite by coupling the OKL with a hierarchical porous nitrogen-doped carbon (NC) [40]. The OKL/NC electrode exhibited good electrochemical properties arising from the synergistic effects of rapid electron transfer, efficient ion diffusion, and a large electroactive surface area. In an acidic solution, the capacitance of the OKL/NC composite (412 F g^−1^ at 1 A g^−1^) was double that of the NC composite (154 F g^−1^).

#### 2.2.2. Conducting Polymer/Lignin Composites

**Figure 6:**
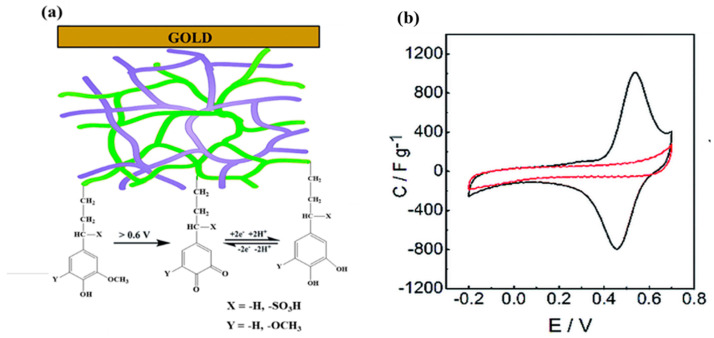
(**a**) Schematic representation of an interpenetrating network of PPy/lignin on an Au substrate, formed through electrochemical polymerization of pyrrole in the presence of lignin. Green lines: lignin with the redox e^−^/H^+^ exchange reaction; purple lines: PPy. (**b**) CV traces of PPy (red) and the PPy/lignin composite (black). Reprinted with permission from ref. [47]. Copyright 2015 Royal Society of Chemistry.

Conducting polymers are suitable hosts for preparing hybrid materials with lignin because of their high electrical conductivities and processabilities, affordability, and electrochemical reversibility [41,42]. Rapid charge transfer occurs in the polymeric chains, with associated counter-ion intercalation and deintercalation during the redox process. Lignin derivates, on the other hand, serve as scalable biopolymer sources to generate Q moieties. The Q/QH_2_ redox reaction has great potential to store charge in lignin/conducting polymer systems. Lignin provides the Q groups in these systems, while the conducting polymer provides electrochemical accessibility to these functional groups [43]. The electrochemical polymerization of pyrrole to polypyrrole (PPy) is feasible in solutions of lignosulfonate and other lignin derivatives [44,45,46]. By combining lignin derivatives with PPy, Milczarek et al. generated an interpenetrating composite appropriate for use as a cathode [44]. Ajjan et al. electropolymerized pyrrole in the presence of a water-soluble lignin derivative (dopant) to prepare PPy/lignin composite electrodes (Figure 6a), then used in situ FTIR spectroelectrochemistry to determine their charge storage mechanism [47]. The formation of Q units, and their reversible redox reactions, occurred during charging/discharging of the electrode materials. A C=O absorption near 1705 cm^−1^ was linked to the creation of Q units during oxidation, whereas that near 1045 cm^−1^ was linked to QH_2_ units. The cyclic voltammogram of the PPy/lignin system exhibited quasi-reversible redox signals corresponding to Q units with a formal potential (*E*′_0_) of 0.57 V (vs. Ag/AgCl) in acid solution; these moieties were derived from the monolignol units present in the lignin (Figure 6b). In contrast to the PPy/lignin system, the PPy/ClO_4_^–^ blend provided a capacitive-like rectangular voltammogram without any pronounced peaks, implying that only rapid insertion/withdrawal of the ClO_4_^−^ anion occurred into/from the polymer. Because it provided an additional Faradaic reaction, the presence of lignin enhanced the total capacity of this system greatly.

**Figure 7:**
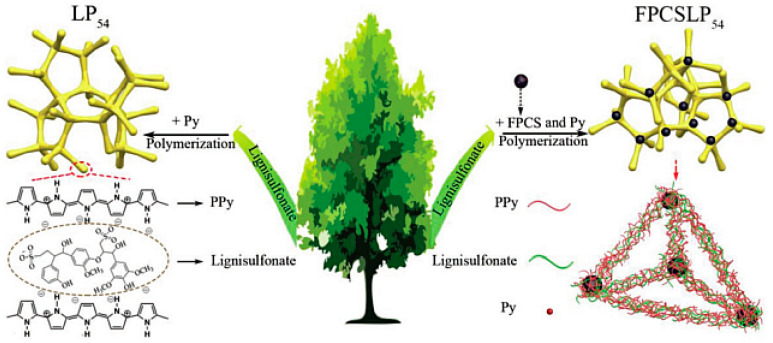
Schematic representation of the preparation of LP_54_ and FPCSLP_54_. Reprinted with permission from ref. [50]. Copyright 2019 John Wiley & Sons.

Admassie et al. added phosphomolybdic acid (HMA, H_3_PMo_12_O_40_**·***n*H_2_O) to the PPy/lignin composite to improve its specific capacitance even further [48]. The inclusion of HMA boosted the specific capacitance of the PPy/lignin composite from 477 to 682 F g^−1^ (at 1 A g^−1^) and greatly enhanced the charge storage capacity (from 69 to 128 mA h g^−1^). Notably, the biopolymer and the inorganic acid dopant both required an acidic electrolyte medium (0.1 M HNO_3_) to ensure that protonation occurred during the reduction process as well as for the development of the reversible redox activities of each dopant. Zhang et al. used in situ polymerization of pyrrole in a lignin/pyrrole solution to develop an integrated, mechanically stiff, all-in-wood supercapacitor featuring a lignin/PPy hydrogel embedded in the wood [49]. Taking advantage of the high pseudocapacitance of lignin, strong interactions between lignin and wood, and the hierarchical porous structure of the wood, featuring vertical channels, the as-prepared integrated lignin/PPy-wood supercapacitor displayed a high areal capacitance (1062 mF cm^−2^), a high energy density (47.2 µW h cm^−2^), and favorable cyclic performance. Peng et al. used oxidative polymerization of pyrrole in the presence of lignin to fabricate a lignin/PPy hydrogel (LP_54_) [50]. To enhance its mechanical strength and electrochemical performance, they introduced functionalized porous carbon nanospheres (FPCS; prepared through the hydrothermal treatment of porous carbon nanospheres) and pyrrole into the prepared LP_54_ system prior to oxidative polymerization of pyrrole, thereby obtaining an FPCS/lignin/PPy hydrogel (FPCSLP_54_, Figure 7). Because of its porous structure and functionalization with pyrrole, the FPCS was anchored and distributed uniformly within the lignin/PPy framework. Although the LP_54_ sample exhibited high specific capacitance (409 F g^−1^ at 0.5 A g^−1^), that of the FPCSLP_54_ hydrogel was even higher (538 F g^−1^) when measured at the same current density. Symmetric flexible supercapacitors constructed using the FPCSLP_54_ hydrogel (electrodes) and a biodegradable cellulose hydrogel (electrolyte separator) exhibited mechanical flexibility and excellent electrochemical properties.

**Figure 8:**
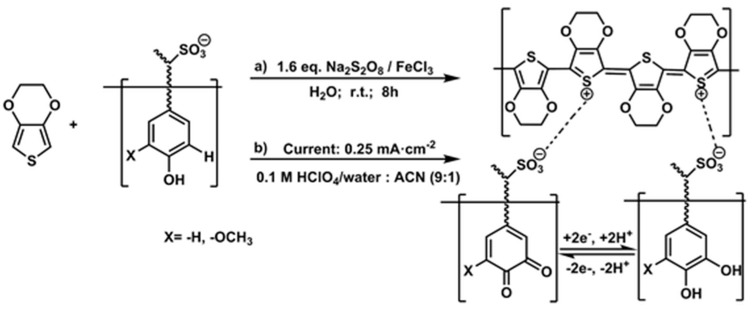
(**a**) Chemical oxidative and (**b**) electrochemical polymerizations of PEDOT/lignosulfonate composites. Reprinted with permission from ref. [51]. Copyright 2016 Royal Society of Chemistry.

Ajjan et al. synthesized PEDOT/lignosulfonate biocomposites by combining lignin with the conducting polymer poly(3,4-ethylenedioxythiophene) (PEDOT) through oxidative chemical and electrochemical polymerizations of EDOT in the presence of lignosulfonate [51]. Although the polymerization of EDOT is simple, poor solubility makes the processing of PEDOT difficult. Polymerizing EDOT in the presence of a water-soluble polyelectrolyte can overcome this problem. Polystyrenesulfonate (PSS) is most typically used, providing a PEDOT:PSS dispersion in water, with PSS serving as both the dopant and dispersing agent. Lignosulfonate, which has sulfonate groups that function similarly to those of PSS, also acts as a dopant and dispersion agent. Indeed, lignosulfonate could disperse EDOT in an aqueous solution, resulting in a PEDOT/lignosulfonate composite after polymerization. The PEDOT/lignosulfonate composite synthesized through chemical and electrochemical polymerizations (Figure 8) were ionic complexes in which the PEDOT polymer and the lignin sulfonate anions interacted to form an interpenetrating polymer network. The specific capacitance of the lignosulfonate-doped PEDOT (170.4 F g^−1^) was double that of the reference PEDOT electrode (80.4 F g^−1^).

Improved charge storage qualities have been achieved when combining redox-active biopolymers with conducting polymers, but their performances have been studied mostly at the electrode level. Navarro-Suárez et al. assembled full-cell supercapacitors featuring positive electrodes made of natural lignin (lignin/PEDOT) and negative electrodes prepared from partially reduced graphite oxide (prGrO) [52]. Combining lignin with PEDOT enhanced its redox characteristics. The asymmetric device had a high cell capacitance (34.6 F g^−1^ at 0.1 A g^−1^). Using PEDOT/lignin as the base layer, and subsequent deposition of polyaminoanthraquinone (PAAQ), Ajjan et al. prepared a hybrid PEDOT/lignin/PAAQ electrode through two-step galvanostatic electropolymerization, then examined its electrochemical properties as a supercapacitor [43]. Hybrid electrode materials constructed from electroactive and conducting components result in supercapacitors possessing intrinsically high specific power and enhanced energy density. The asymmetric supercapacitor fabricated using PEDOT/lignin/PAAQ (positive electrode) and PEDOT/PAAQ (negative electrode) exhibited superior specific capacitance (74 F g^−1^) as a result of the synergistic effects of its two electrodes.

**Figure 9:**
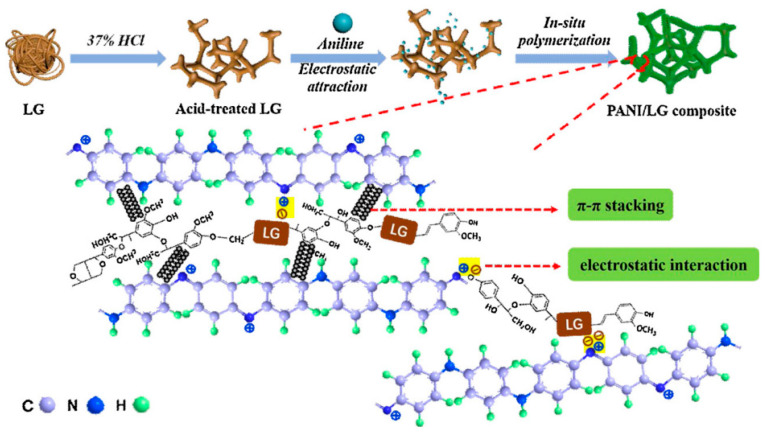
Schematic representation of the mechanism of formation of the PANI/lignin composite and the possible interactions among its components. Reprinted with permission from ref. [53]. Copyright 2019 Elsevier.

Wang et al. performed in situ oxidative polymerization of aniline with reticulated lignin, yielding polyaniline (PANI)/lignin composites possessing interpenetrating fibrous networks (Figure 9). [53]. In acidic solutions, aniline mostly forms cations, according to the polymerization mechanism. Because of its aromatic OH groups and CH_2_OH groups, lignin can be negatively charged in slightly acidic solutions. When combined, aniline cations can adsorb electrostatically onto the surface of lignin. Upon the introduction of ammonium persulfate (APS), aniline cations attached to the surface of reticulated lignin, which underwent oxidation to aniline cationic radicals, which reacted through radical polymerization to form PANI/lignin composites. These composites outperformed PANI itself as electrodes for supercapacitors, with respect to electrochemical capacitance, rate capability, and cycle stability. At 30 A g^−1^, the PANI/lignin composite provided a specific capacitance of 284.4 F g^−1^ and a capacitance retention of 58.6% when the current density was increased from 0.5 to 30 A g^−1^; for PANI these values were 123.1 F g^−1^ and 29.2%, respectively. Furthermore, at 1.0 A g^−1^, the capacity retention of the PANI/lignin symmetrical capacitor was 67.4% after 5000 cycles, outperforming PANI (17.8%).

**Figure 10:**
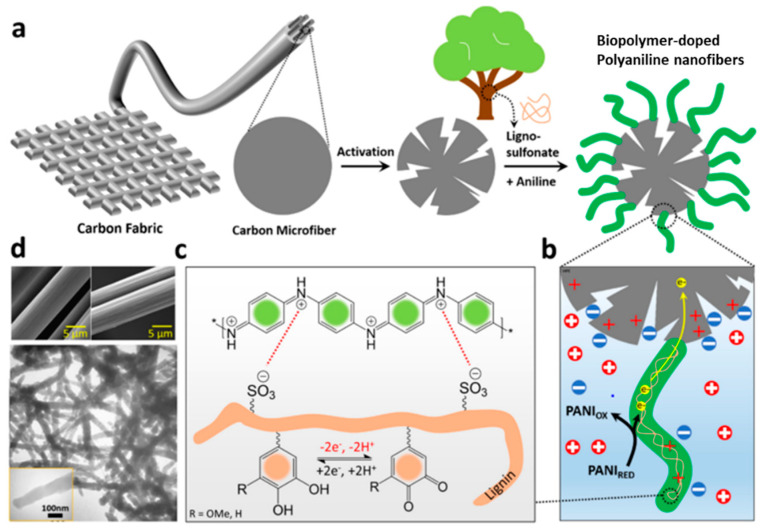
(**a**) Schematic representation of electrochemical etching of a carbon microfiber under a constant potential of +2.0 V for 180 s in 0.5 M H_2_SO_4_ and subsequent electro-polymerization of aniline in the presence of lignosulfonate chains on the electrochemically etched carbon fiber substrate. (**b**) Faradaic charge transfer and electrical double-layer charge accumulation at the electrode–electrolyte interface. (**c**) Electrochemical activity of lignosulfonate moieties as dopants and structure-directing agents for PANI films, and the redox activity of lignosulfonate (forming catechol moieties) in PANI/lignin nanocomposites. (**d**) (top) SEM images of the carbon fiber substrate (left) before and (right) after electrochemical etching; (bottom) TEM image of the resulting PANI/lignin nanocomposite. Reprinted with permission from ref. [54] Copyright 2021 American Chemical Society.

The types of counter-anions used to compensate for the positive charges of the conjugated polymer chains during synthesis can greatly impact the morphological and structural properties, as well as the electrochemical behavior, of the conducting polymers. Small anionic dopants (e.g., Cl^−^, ClO_4_^−^, *p*-toluenesulfonate, and dodecylbenzenesulfonate) are prone to migrate out of the electrodes, whereas the use of large (bio)polymers as dopants can result in confinement of the redox moieties within the matrix of the conductive polymer. Dianat et al. employed an improved method for the preparation of a nanocomposite featuring an interpenetrating PANI/lignin network on an electrode of electrochemically etched carbon fiber [54]. They obtained a uniform PANI/lignin film after applying a pattern of potential pulses to regulate the nucleation kinetics and growth behavior (Figure 10). This PANI/lignin nanocomposite outperformed the best-known conducting polymer/lignin supercapacitors by delivering a specific capacitance of 1200 F g^−1^ at 1 A g^−1^, based on rapid H^+^ insertion/de-insertion kinetics, rather than sluggish SO_4_^2−^ doping/dedoping processes. The PANI and H_2_Q/Q couples responsible for the charge storage mechanism in PANI/lignin nanocomposite electrodes can be represented as follows:

(2) $${\mathrm{PANI}}_{0X}+2H^{+}+2e^{-}⇄{\mathrm{PANI}}_{\mathrm{Red}}$$

(3) $${\mathrm{PANI}}_{\mathrm{Red}}+\mathrm{Lignin}-Q⇄{\mathrm{PANI}}_{0X}+\mathrm{Lignin}-H_{2}Q$$

(4) $$\mathrm{Lignin}-Q+2H^{+}+2e^{-}⇄\mathrm{Lignin}-H_{2}Q$$

with the overall reaction expressed as

(5) $${\mathrm{PANI}}_{0X}.\mathrm{Lignin}-Q+4H^{+}+4e^{-}⇄{\mathrm{PANI}}_{\mathrm{Red}}.\mathrm{Lignin}-H_{2}Q$$

Because the SO_3_^−^ dopants on lignosulfonate cannot migrate from the polymer, charges must be balanced through insertion of H^+^ cations into the polymer. The transport of small H^+^ cations in and out of the PANI/lignin matrix is more efficient than that of bulkier SO_4_^2−^ anions, which mediate charge compensation during the redox reactions of pure PANI electrodes. A symmetric PANI/lignin||PANI/lignin device offered high specific energy (21.2 W h kg^−1^) and exceptional specific power (26.0 kW kg^−1^), as well as remarkable cycle stability and flexibility.

Tanguy et al. used faradaic reactions of Q groups in natural lignin, covalently attached to high-strength cellulose nanofibrils, to construct mechanically strong and flexible thin film electrodes displaying good energy storage capability [55]. To fabricate the flexible electrodes, they grew PANI on flexible films made of lignin-containing cellulose nanofibrils (LCNFs) and RGO nanosheets at various loadings. The LCNF/RGO/PANI electrode containing 20 wt.% RGO nanosheets displayed specific capacitances (475 F g^−1^ at 10 mV s^−1^; 733 F g^−1^ at 1 mV s^−1^) up to 68% greater than those of comparable electrodes constructed without lignin. The electrochemically active LCNFs performed multiple functions, providing the composite electrode with mechanical strength and flexibility, while improving the overall energy storage. The presence of the lignin and Q/QH_2_ units on the surface of the LCNFs presumably resulted in additional π–π interactions with the PANI and RGO nanosheets in the films, helping to increase the specific capacitance of the produced electrodes.

#### 2.2.3. Metal Oxide/Lignin Composites

Because of their attractive structural and electrochemical characteristics, metal oxides (e.g., MnO_2_, CoO, V_2_O_5_, RuO_2_, NiO, and CuO) and their composites have been applied widely for supercapacitors [56,57,58]. The ability to attain relatively high pseudocapacitive performance, arising from their many valence state transitions, is key to their electrochemical characteristics [59]. Combining lignin with metal oxides can improve the electrochemical characteristics of electrodes [60,61,62,63,64]. Incorporating lignin into metal oxides for supercapacitor applications has proven challenging, due to difficulties in regulating the resulting electrochemical properties, with significant impacts on the cycle life and performance of the devices. As a result, only a few studies have looked at mixing metal oxides with lignin to take advantage of their individually attractive properties. Jha et al. fabricated an anode by depositing NiWO_4_ nanoparticles (NPs), coated with bio-derived alkali lignin, onto an aluminum substrate for use as a supercapacitor [63]. To determine the proportions of these components which provides the greatest electrochemical performance, they examined a variety of lignin:NiWO_4_:poly(vinylidene difluoride) (PVDF) compositions: 80:10:10, containing mostly lignin; 45:45:10, having equal levels of lignin and NiWO_4_; and 10:80:10, featuring mostly NiWO_4_ NPs. A larger fraction of NiWO_4_ NPs in the supercapacitor, relative to lignin, resulted in better specific capacitance and retention. A supercapacitor fabricated using an Al/lignin–NiWO_4_ anode, an Al/AC cathode, and PVA/H_3_PO_4_ as the gel electrolyte had a specific capacitance of 17.01 mF cm^−2^ at 0.13 A g^−1^, good stability (84% capacitance retention after 2000 cycles), a maximum energy density of 2 W h cm^–2^, and a maximum power density of 100 W cm^−2^. Jha et al. also prepared NiCoWO_4_-decorated lignin electrodes for flexible supercapacitors and investigated the effect of Ni as a secondary metal on pseudocapacitance of NiCoWO_4_ [60]. Electrochemical testing of the lignin/NiCoWO_4_/AC- and lignin/CoWO_4_/AC-based supercapacitors revealed that the specific capacitance of the bimetallic tungstate (NiCoWO_4_)-containing supercapacitor (862.26 mF cm^−2^) was 141 times higher than that of the monometallic tungstate (CoWO_4_)-containing supercapacitor. Because of the synergistic effect of the bimetallic tungstate NPs encapsulated in the lignin, the lignin/NiCoWO_4_ supercapacitor also possessed an exceptionally high power density (854.76 kW kg^−1^) and energy density (5.75 W h kg^−1^). Moreover, Jha et al. also studied the electrochemical performance of carbonized lignin/NiCoWO_4_ on a graphene cathode, to identify the effects of smaller fragments of lignin, and partially oxidized lignin, on the capacitance performance. The specific capacitance of the carbonized lignin/NiCoWO_4_ was inferior to that of the non-carbonized lignin/NiCoWO_4_. During carbonization, the long carbon chains of lignin were broken down into smaller fragments. To some extent, oxidation occurred. As a result of the higher impedance to charge transfer resulting from fragmentation of the lignin chains, and related irregularity in the chain structure and arrangement, the carbonized lignin exhibited decreased capacity to store charge.

Jha et al. used a similar approach to fabricate Al/lignin/MnO_2_–based anodes for supercapacitors [61]. They decorated alkali lignin with MnO_2_ particles and then coated it onto an Al substrate to make the composite electrode. To build the supercapacitor, an Al/lignin/MnO_2_ anode and an Al/AC cathode were sandwiched together with a polymer gel-type electrolyte (PVA/H_3_PO_4_). Lignin/MnO_2_ blends of various ratios were used to optimize the electrode performance. The assembled supercapacitor had a high specific capacitance (379 mF cm^−2^ at 40 mA g^−1^), good cycling stability (80% retention of capacitance), and maximum energy and power densities of 6 W h kg^−1^ and 355 W kg^−1^, respectively. Later, Jha et al. deposited MnO_2_ hydrothermally onto AC and lignin to create an asymmetric supercapacitor [62]. The supercapacitor, which featured an anode of Al/AC/lignin-MnO_2_, a cathode of Al/AC, and a gel electrolyte of PVA/H_3_PO_4_, exhibited a specific capacitance of 5.52 mF cm^−2^ at 6.01 mA g^−1^. Mehta et al. employed microwave irradiation to create micro-MnO_2_ particles, which they subsequently deposited hydrothermally onto Al/lignin and Al/AC/lignin substrates [64]. The specific capacitance of the AC/lignin-MnO_2_–based supercapacitor was greater than that of that based on lignin-MnO_2_, because of the high surface area and porosity of AC.

## 3. Lignin-Based Electrolyte Materials for Supercapacitors

Redox-active electrolytes (REs) are alternative electrolytes displaying great potential for enhancing the specific capacitance of supercapacitors by contributing pseudocapacitance from the reduction/oxidation of the redox mediator in the electrolyte [65]. In other words, both the electrolyte and the pseudocapacitive electrode contribute to the pseudocapacitance. A number of redox mediators—including Cu^2+^/Fe^2+^ [66], VO^2+^/VO_2_^+^ [67], Fe^3+^/Fe^2+^ [68], KI [69,70], potassium ferricyanide K_3_(Fe(CN)_6_) [71,72], K_4_(Fe(CN)_6_) [71], and Na_2_MoO_4_ [73]—have been introduced into liquid electrolytes to enhance the capacitance of carbon-based supercapacitors. Several organic redox mediators have also been studied as pseudocapacitive sources in supercapacitors, including QH_2_ [74,75,76], methylene blue [77], *p*-benzenediol [78], *p*-phenylenediamine [76,79], *m*-phenylenediamine [80], humic acids [81], indigo carmine [82], lignosulfonates [83], and sulfonated polyaniline [84].

Lignin has been examined as an electrolyte additive to allow reversible electron saving/extraction in Q-type moieties. Through faradaic reactions occurring at porous electrode/electrolyte interfaces, lignin can efficiently boost the capacitance of supercapacitors. Lota et al. studied the effect of addition of a lignosulfonate in the electrolyte on the electrochemical performance of supercapacitors [83]. The presence of the lignosulfonate as an electrolyte additive improved the total capacitance of the supercapacitor by up to 33%, according to measurements of two-electrode cells. Three-electrode experiments revealed that adding lignosulfonate to the electrolyte resulted in a reversible redox system developing on the positive electrode, arising from deposition of a thin lignosulfonate-derived film with substantial redox activity, attributed to Q-type units.

The use of lignin and its derivatives to make gel electrolytes for flexible energy storage devices has also been investigated. Park et al. used base-catalyzed ring-opening polymerization (ROP) and chemical cross-linking to create lignin hydrogel electrolytes [85]. Because of their mechanical and dimensional stabilities and the large amount of water they could uptake as a result of their cross-linking chemistry, these cross-linked lignin hydrogel electrolytes were mechanically robust (swelling ratio: 523%) and displayed high ionic conductivity (10.35 mS cm^−1^). A renewable flexible supercapacitor incorporating the chemically cross-linked lignin hydrogel electrolyte and electrospun lignin/polyacrylonitrile-derived nanofiber electrodes exhibited high capacitance, flexibility, and durability when bent at various angles, and retained 95% capacitance over 10,000 cycles. The maximum specific capacitance of the supercapacitor electrode incorporating the lignin hydrogel electrolyte (129.23 F g^−1^) was greater than those of PVA/KOH gel electrolytes at 0.5 A g^−1^ (68.48 F g^−1^ for 1.0 M KOH; 104.09 F g^−1^ for 3.3 M KOH).

**Figure 11:**
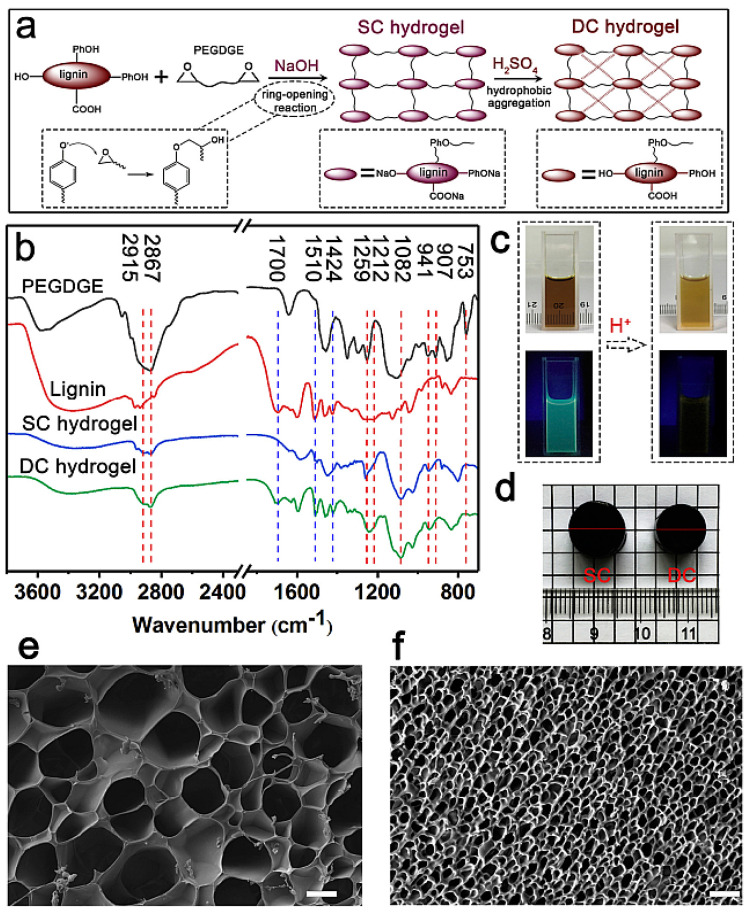
(**a**) Schematic representation of double-crosslinking reaction of DC lignin hydrogel through chemical crosslinking polymerization, followed by physical hydrophobic aggregation crosslinking. (**b**) FTIR spectra of poly(ethylene glycol) diglycidyl ether (PEGDGE), lignin, and the single-network (SC lignin hydrogel) and double-network (DC lignin hydrogel) lignin hydrogels. (**c**) Photographs of lignin in NaOH solution before and after treatment with H_2_SO_4_: (top) bright field images; (bottom) irradiated at 365 nm. (**d**) Photographs of the SC and DC lignin hydrogels. (**e**,**f**) SEM images of the (**e**) SC and (**f**) DC lignin hydrogels; scale bars: 20 µm. Reprinted with permission from ref. [86]. Copyright 2020 Elsevier.

A double-crosslinking strategy based on two networks has improved the mechanical characteristics of hydrogels. The first network, which was rigid and brittle, acted as a sacrificial bond, efficiently dissipating energy; the second network, which was soft and ductile, kept the hydrogel intact during deformation. Liu et al. reported a new double-crosslinked lignin hydrogel electrolyte possessing excellent compressibility (Figure 11) [86]. Because of its high phenol content, they chose enzymatically hydrolyzed lignin (*M*_w_ = 3000–5000 Da) to create a highly chemically crosslinked network. First, a chemically crosslinked lignin hydrogel (SC lignin hydrogel) was synthesized using base-catalyzed ROP and crosslinking; simply soaking it in acid converted the SC lignin hydrogel into a highly mechanically stable hybrid double-crosslinked lignin hydrogel (DC lignin hydrogel). Immersion of the SC lignin hydrogel in 1 M H_2_SO_4_ protonated any free phenol and carboxyl groups within the lignin, resulting in hydrophobic interactions (physical crosslinking) between lignin chains. The resulting DC lignin hydrogel has a compressive mechanical strength of 4.74 MPa, 40 times higher than that of the SC lignin hydrogel, as well as outstanding cyclic loading/unloading compression performance. Furthermore, the ionic conductivity of this type of DC lignin hydrogel electrolyte was extremely high (0.08 S cm^−1^), equivalent to that of pure H_2_SO_4_. Therefore, when Liu et al. constructed a supercapacitor incorporating this synthetic DC lignin hydrogel as the electrolyte, the device had a remarkable specific capacitance (190 F g^−1^), an excellent rate capability, and a high energy density (15.24 W h kg^−1^)—performance characteristics similar to, and in some cases greater than, those of other flexible supercapacitors.

In general, increases in specific capacitance by approximately 2–4 times have been observed for carbon-based supercapacitors when adding these organic redox additives to the electrolyte [73,87]. Notably, migration between the electrodes of a redox-active species in the electrolyte often resulted, however, in much more rapid self-discharging of the supercapacitors when using a QH_2_-based redox-active electrolyte [87].

## 4. Summary

Lignin, a renewable bioresource, is a very attractive material for use in high-performance supercapacitors because of its low cost and environmentally benign character. This review summarizes recent developments in the fabrication of lignin-based electrode materials and electrolytes applied in supercapacitors. The phenol groups in lignin and its derivatives can form redox-active Q/QH_2_ structures. As summarized in Table 1, early studies revealed that lignin and its derivatives could function as effective supercapacitor electrodes when combined with electrically conducting substances. Lignin may also be used as a redox-active additive in supercapacitor electrolytes. The inclusion of lignin in the electrode materials and electrolytes of supercapacitors not only enhances their performance but also lowers their cost and toxicity, resulting in greener supercapacitor devices.

Table 1 shows that only a few lignin sources, such as kraft lignin and lignosulfonate, have been studied for use in supercapacitors. As a result, it is essential to conduct in-depth study on lignin products from diverse plant species in different biorefinery processes for applications in supercapacitors.

## 5. Challenges and Future Perspectives

It has been successfully demonstrated in prior studies that lignin and its derivatives may be used as redox electrolytes and electrode materials for supercapacitors. However, much more research will be necessary to realize lignin-based electrode materials for practical energy storage applications. Here we provide a summary of the current difficulties, and potential prospects, of lignin-based electrode materials and electrolytes.

- Lignin is a chemical extracted from biomass. During biomass pulping, cellulose recovery has had priority over lignin recovery. As a result, instead of favoring the production of lignin, pulping procedures are tailored to extract high-quality cellulose. The result is a somewhat impure lignin byproduct that contains contaminants from upstream processes. This impure lignin of high calorific value has generally been burned to generate heat and power in the pulp and paper industries. Therefore, improved lignin-first recovery strategies for the extraction and application of high-purity lignin will be critical for future developments.
- Technical lignins exhibit chemical and structural heterogeneity depending primarily on the plant source and the nature of the extraction method. Therefore, it will be necessary to develop efficient methodologies for the fractionation of lignin to address the technical issues associated with lignin heterogeneity, in terms of chemical compositions and molecular weight distributions. Moreover, a detailed and methodical research of the various lignin types is required to understand how differing chemical structures may change the electrochemical activity of lignin.
- Since lignin is electrically insulating, accessing the redox active Q-groups requires conductive materials. Lignin has been coupled with a number of expensive materials, such as conductive polymers, metal oxides, and carbon materials, to form hybrid materials for supercapacitors. Considering the cost reduction for the large-scale production of supercapacitors, finding compatible and cost-effective conductive materials to synergistically interact with lignin to create hybrid electrodes is highly desirable.
- Lignin-based pseudocapacitive materials are susceptible to rapid self-discharge. A high self-discharge rate results in rapid loss of energy and power density. To reduce the self-discharging rate, it is crucial to investigate the self-discharge mechanisms of lignin-based hybrid electrodes.
- Because of the relatively fixed amounts of QH_2_ species in lignin, increasing the pseudocapacitance as a result of the natural lignins themselves, undergoing Q/QH_2_ redox cycling, remains difficult. Therefore, achieving greater pseudocapacitances would ideally occur through the development of functionalized modification techniques to selectively cleave ether bonds between pairs of propylphenyl units, thereby generating additional phenolic OH and QH_2_ groups.
- It is necessary to conduct a more thorough techno-economic evaluation of novel lignin-derived products to pinpoint process bottlenecks and guide future research paths. Performance-to-price considerations are always crucial for the practical application of lignin in supercapacitors.

Although significant improvements have been made in the performance of lignin-derived materials, as well as in our understanding of the mechanisms responsible for their behavior in supercapacitors, many obstacles will need to be overcome before lignin can be used commercially in supercapacitors. We hope that continued studies by the scientific community will provide additional intriguing breakthroughs and—eventually—viable, high-value, lignin-derived, sustainable supercapacitors.

## Figures and Tables

**Table 1:** Overview of lignin-based electrodes for supercapacitors.

Electrode Material	Lignin Type	Electrolyte	Specific Capacitance	Cycle Life	Reference
RGO–lignin composite	Softwood sodium lignosulfonate	0.1M HClO_4_	432 F g^−1^ at 10 mV s^−1^	∼96 % after 3000 cycle	[30]
Lignin-RGO composite	Alkali lignin	6 M KOH	190 F g^−1^ at 0.5 A g^−1^	86.5% after 10,000 cycles	[32]
Lignin/graphene hydrogels	Ligninsulfonate	0.1 M HClO_4_	549.5 F g^−1^ at 1 A g^−1^	83.7% after 1000 cycles	[29]
Graphene–lignin composite	Soda bagasse lignin	0.1 M HClO_4_	211 F g^−1^ at 1.0 A g^−1^	88% after 15,000 cycles	[33]
Lignosulfonate-functionalized graphene hydrogels	Ligninsulfonate	1 M H_2_SO_4_	432 F g^−1^ at 1 A g^−1^	98.8% after 2000 cycles	[34]
CNT/ lignin composite	Kraft lignin	1 M H_2_SO_4_	143 F g^−1^ at 50 mV s^−1^	93% after 500 cycles	[31]
Lignin/single-walled CNT hydrogel	Ligninsulfonate	Cellulose/Li_2_SO_4_ gel	292 F g^−1^ at 0.5 A g^−1^	80.1% after 10,000 cycles	[38]
Kraft lignin-modified HNO_3_-treated active carbon	Kraft lignin	1 M H_2_SO_4_	293 F g^−1^ at 1 A g^−1^	98.1% after 1000 cycles	[39]
Oxidized Kraft lignin (OKL)/TAC composite	Kraft lignin	1 M H_2_SO_4_	390 F g^−1^ at 0.5 A g^−1^	97.9% after 2000 cycles	[35]
OKL/hierarchical porous nitrogen-doped carbon	Kraft lignin	1 M H_2_SO_4_	412 F g^−1^ at 1 A g^−1^	93.8% after 1000 cycles	[40]
PPy/lignin composite	Ligninsulfonate	0.1 M HNO_3_	682 F g^−1^ at 1 A g^−1^	70% after 2000 cycles	[48]
Lignin/PPy hydrogel	Sodium Lignosulfonate	1 M H_2_SO_4_	1062 mF cm^−2^ at 1 mA cm^−2^	82.1% after 5000 cycles	[49]
FPCS/lignin/PPy hydrogel	Sodium Lignosulfonate	Cellulose/H_2_SO_4_ hydrogel	538 F g^−1^ 1 at 0.5 A g^−1^	79.8% after 7000 cycles	[50]
PEDOT/lignosulfonate biocomposite	Sodium Lignosulfonate	0.1 M HClO_4_/water : acetonitrile (9 : 1) mixed solvent	170.4 F g^−1^ at 1 A g^−1^	80% after 1000 cycles	[51]
Lignin/PEDOT	Kraft lignin	0.1 M HClO_4_	97 F g^−1^ at 0.1 A g^−1^	79% after 1000 cycles	[52]
PEDOT/lignin/PAAQ	Sodium Lignosulfonate	0.1 m HCLO_4_	418 F g^−1^ at 1 A g^−1^	100% after 5000 cycles	[43]
PANI/lignin composite	Kraft lignin	1.0 M HClO_4_	284.4 F g^−1^ at 0.5 A g^−1^	67.4% after 5000 cycles	[53]
PANI/lignin nanocomposite	Sodium Lignosulfonate	0.5 M H_2_SO_4_	1200 F g^−1^ at 1 A g^−1^	87% after 15,000 cycles	[54]
LCNF/RGO/PANI	1% NaOH treated red cedar bark	0.5 M H_2_SO_4_	475 F g^−1^ at 10 mV s^−1^	87 % after 5000 cycles	[55]
Al/lignin–NiWO_4_	Kraft lignin	PVA/H_3_PO_4_	17.01 mF cm^−2^ at 0.13 A g^−1^	84% after 2000 cycles	[63]
Lignin/NiCoWO_4/_AC	Kraft lignin	PVA/H_3_PO_4_	862.26 mF cm^−2^	100% after 2000 cycles.	[60]
Al/lignin/MnO_2_	Kraft lignin	PVA/H_3_PO_4_	379 mF cm_−__2_ at 40 mA g^−^^1^	80% after	[61]
Al/AC/lignin-MnO_2_	Kraft lignin	PVA/H_3_PO_4_	5.52 mF cm^−^^2^ at 6.01 mA g^−^^1^	97.5% after 2000 cycles	[62]
AC/lignin-MnO_2_	Kraft lignin	PVA/H_3_PO_4_	22 mF cm^−2^ at 10 mV s^−1^	90% after 700 cycles	[64]

## Data Availability

Not applicable.
